# Selective Sensing of Mixtures of Gases with CMOS-SOI-MEMS Sensor Dubbed GMOS

**DOI:** 10.3390/mi14020390

**Published:** 2023-02-04

**Authors:** Adir Krayden, Dima Shlenkevitch, Tanya Blank, Sara Stolyarova, Yael Nemirovsky

**Affiliations:** 1 Electrical and Computer Engineering Department, Technion—Israel Institute of Technology, Haifa 3200003, Israel; 2 Todos Technologies, Kinneret 12 Street, Airport City 7019900, Israel

**Keywords:** MEMS, gas sensor, SOI, MOS, data analytics, classification, machine learning, tinyML, Kalman filtering

## Abstract

The need to achieve digital gas sensing technology, namely, a technology to sense and transmit gas-enabled digital media, has been recognized as highly challenging. This challenge has motivated the authors to focus on complementary metal oxide semiconductor silicon on insulator micro electro-mechanical system (CMOS-SOI-MEMS) technologies, and the result is a new pellistor-like sensor, dubbed GMOS, with integrated signal processing. In this study, we describe the performance of such sensors for the selective detection of mixtures of gases. The novel key ideas of this study are: (i) the use of the GMOS for gas sensing; (ii) applying the Kalman filter to improve the signal-to-noise ratio; (iii) adding artificial intelligence (AI) with tiny edge approach.

## 1. Introduction

In recent years, the need for mobile, low cost, and low power gas sensors has increased dramatically. Such gas sensors are needed for safety in homes and cars, monitoring air quality, the wellbeing of people, as well as industrial process control and precision agriculture. In practice, all these applications require data logging and, hence, digital sensing systems become essential.

The need to achieve digital gas sensing technology, namely, a technology to sense and transmit gas-enabled digital media (such as pictures and music) has been recognized as highly challenging. This challenge has motivated the authors to focus on CMOS-MEMS (micro electro-mechanical system) technologies, and the result is a new pellistor-like sensor, dubbed GMOS. Since the first report in 2018, several papers describing GMOS have been reported by the same group [1,2,3,4,5,6].

The main uniqueness of the GMOS sensor is its ability to detect selectively different gases. Selectivity is achieved by measuring a fundamental physical property—heat of exothermic reaction and ignition temperature, instead of measuring relative changes of resistance, as in metal oxide semiconductor gas sensors [1]—thus enabling selectivity (see Section 3).

Currently, no commercial devices, apart from bulky and expensive gas chromatographs, can selectively detect ethanol and acetone in mixtures. The compact commercial ethanol and acetone sensors are, in fact, the TVOC (total volatile organic compound) sensors that detect all these compounds together, for example, SPG40—Sensirion [7], ZMOD4410—Renesas [8], based on MOX chemiresistors, and MiniPID 2WR—Ionsense, based on the photo-ionization principle [9]. Among the research papers, there were few attempts to measure ethanol selectively, however, the selectivity of ethanol measurement was not evaluated relative to acetone [10,11]. Table A1 compares GMOS with the commercial sensors. The values are taken from the datasheets of the commercial products. 

In real-life applications, there is an even more challenging task: to selectively measure mixtures of gases. This study focuses on applying the Kalman filter [12,13,14,15,16,17] to improve the signal-to-noise ratio, and subsequently, to add tiny edge machine learning to enable better analysis of gas mixtures. 

Section 2 describes the role of the CMOS-SOI-MEMS technology towards high-performance infra-red (IR) sensing systems based on thermal sensors. Section 3 describes the role of the CMOS-SOI-MEMS technology towards a digital gas sensing system. Section 4 describes the role of the Kalman filter in improving the signal-to-noise ratio. Finally, the tiny edge revolution [18,19], which makes the GMOS smarter by applying machine learning (ML) techniques based on the tiny edge ML, is briefly described.

## 2. The Role of CMOS-SOI-MEMS Fabrication Technology for Uncooled Thermal Sensors

Since the 1980s, the CMOS (complementary metal oxide semiconductor) has remained the dominant microelectronic technology for well established advantages. CMOS technology maintains the essential advantages of low power consumption, high noise tolerance, wide operating voltage, and operating temperature range. Furthermore, it rapidly and continuously improves the integration degree and intrinsic speed. Therefore, CMOS technology has become the most important technology in VLSI (very large scale integration) [20]. 

CMOS MEMS (micro-electric mechanical systems) are micro-machined systems in which MEMS devices are integrated with CMOS circuitry on a single chip to enable miniaturization and performance improvement. With the advancement of both CMOS and micromachining technologies, CMOS MEMS have also evolved tremendously in recent years [21].

The CMOS-SOI-MEMS technology, which includes a buried oxide layer (SOI), enables wafer level processing and packaging in a standard CMOS-SOI FAB. Metallization layers provide built-in masks for MEMS processing. The back-side handle is removed by deep reactive ion etch (DRIE), and the buried oxide (BOX) layer provides an etch stop. Front-side dielectric layers are removed by RIE, while front-side metal masks are removed by etching. With this method, a thermally insulated transistor is achieved. The CMOS-SOI-MEMS unique technology provides good alignment, which is determined by CMOS-SOI technology (1 μm in the present study), and there is no need for expensive masks. This technology has already enabled thermal IR sensors, dubbed digital TMOS (thermal MOS) [22,23]. The excellent performance of digital TMOS in terms of linearity, reproducibility, and accuracy, while achieving low-power and low-cost performance, is also attributed to the CMOS-SOI-MEMS technology. Extending this approach to digital gas sensing (dubbed GMOS) is currently under extensive development by the authors [1,2,3,4,5,6].

## 3. GMOS Design and Operation Principles Based on CMOS-SOI-MEMS

The key element—GMOS multi-gas sensor—is a product of modern VLSI microelectronics, MEMS, nanomaterials, computer algorithms, machine learning, and wireless communication. The GMOS design and operating principles are described in detail in references [1,2,3,4,5,6]. Here, we review the innovation and the unique property of selectivity. 

The breakthrough innovation is in implementation of the suspended MEMS-TMOS transistor for detection of very low gas concentrations. The transistor is integrated with a reaction micro-hotplate covered by catalytic nanoparticles on which exothermic combustion reaction of gas molecules takes place. Such a transistor can detect tiny changes in temperature caused by the exothermic gas reaction. As a result, our sensor demonstrates very high sensitivity and the ability to detect sub-ppm concentration of ethylene, ethanol, and other gases. 

Exploiting this fact, that each gas has a specific ignition temperature, and measuring signal/temperature characteristics, our sensor demonstrates excellent selectivity to ethylene in the presence of other gases, which makes it competitive over all existing commercial gas sensors. Fabricated using matured VLSI technology, GMOS offers low price and consumes low power. 

## 4. Application of Kalman Filter to Improve the Signal to Noise Ratio of the GMOS—Illustrating Experimental Results

### 4.1. Introduction to Estimation Theory and the Kalman Filter

Noise is an integral part of life, which sets a fundamental limit on all electronic circuits and systems operation. Most of the processes are random and not deterministic. Random processes and noise in general are pattern-less, but not property-less [24]. Hence, one good method is the use of estimation theory to analyze data measurements taken from a system.

In particular, optimal estimation theory deals with the development of optimal estimators or algorithms to provide an updated optimal estimate of the system’s state being observed using new measurements (data) [12,16]. 

The Kalman filter is one of the most important estimation algorithms. It helps produce estimations of the state of the system. Additionally, it may be applied for both static and dynamic systems (whereas the system changes or not over time), as well as both stationary and nonstationary environments. The Kalman filter approach includes the generalization of the system into a mathematical model to obtain the optimal state, enabling the prediction of the future system based on past estimations and reducing the noise in the measured value. Generally, the algorithm has a smoothing effect on the system’s measurements, which converge toward the true value. The algorithm requires only the last estimation for each iteration, thus minimizing memory requirements, which also makes the filter suited for real-time systems [12,13,14,15,16,17]. 

GMOS, our gas sensor, changes I–V characteristics based on the gas reactions. Our given signal (i.e., voltage measurement) is noisy (electrical circuits, the sensor itself, etc.). The Kalman filter may help provide more accurate and precise real-time estimations, taking the different random noises into account. 

This paper focuses on improvements, which were made to GMOS, but a brief, simple mathematical explanation will be given. There are several fundamental equations that define Kalman filters.

As each time sample is taken, the state update equation defines the filtered output.

(1) $$\{\begin{array}{c} {\hat{X}}_{n}={\hat{X}}_{n-1}+K_{n}\cdot \left(Z_{n}-{\hat{X}}_{n-1}\right)=\left(1-K_{n}\right)\cdot {\hat{X}}_{n-1}+K_{n}\cdot Z_{n}\\ 0\leq K_{n}\leq 1 \end{array}$$

The equation states that the new filter estimation, (${\hat{X}}_{n}),$ is the previous one, (${\hat{X}}_{n-1})$, plus the difference between the new measurement, ($Z_{n}),\;$and the previous estimate, multiplied by a weight factor, $K_{n}$ (‘Kalman gain’). Generally, the Kalman gain tends to be higher when the measurement uncertainty (the variance of the measurements errors, the differences between the measurements, and the true values) is low and the estimate uncertainty (estimation of the difference between the true values and the filter estimates) is high, and vice versa. Usually, Kalman gain tends to decrease as the quantity of the measurements increases. The filter, therefore, ‘prefers’ new measurements at the start, but as the estimate becomes more accurate, new measurements have less impact (if the estimate does not match the measured values, the Kalman gain will become larger). We also used the following equations:

(2) $$\{\begin{array}{c} K_{n}=\frac{p_{n-1}}{p_{n-1}+r}\\ p_{n}=\left(1-K_{n}\right)\cdot p_{n-1}+\left|Z_{n}-{\hat{X}}_{n}\right|\cdot q \end{array}$$

where $p_{n-1}$ is the extrapolated estimate uncertainty, r is the measurement uncertainty, and *q* is the process noise. The estimate error represents the difference between the true values and the estimates. On the contrary, the measurement uncertainty, r, represents the difference between the true values and the measurements. The measurement error is random, hence, it may be described by a variance. The parameter *q* represents the uncertainty of the model (system noise). Both *q* and r are given as inputs to the algorithm. 

One should know that the filter performance also depends on the input noise parameters (*q* and *r*). If the latter are wrong, the accuracy of the filtering might be reduced. Furthermore, inaccurate estimations may continue to accumulate, therefore, the user will continue to receive inaccurate results. 

### 4.2. Experimental Results

**Figure 1:**
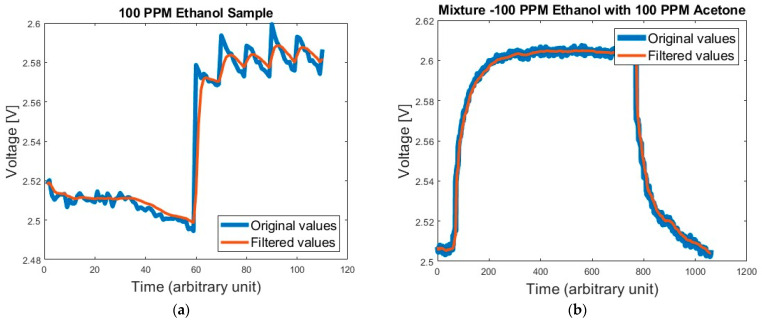
Examples to the readout improvements made by the Kalman filter. The original data and the filtered data may be seen. The filtered output is smoother and more resistant to noise. (**a**) 100 PPM Ethanol. (**b**) A mixture of 100 PPM ethanol and 100 PPM acetone.

As explained above, Kalman filter helps improve the SNR (signal-to-noise ratio), as may be seen in Figure 1.

As may be seen, after applying the Kalman filter, the output is smoother and less affected by noise. The filtered output might have a slight delay (of the order of ~2 s) compared to original data. Even so, those delays do not harm GMOS performance.

## 5. TinyML for Gas Classification

Machine learning (ML) is the use of algorithms that are capable of learning and adapting. The resource-intensive nature of ML is one of its major problems. For neural networks, for instance, large training sets and great computer power are usually required. Thus, there is a potential for ML solutions in small and resource-constrained systems and edge devices [18]. An emerging subfield of machine learning called tinyML, combines embedded systems with machine learning. Devices can run ML applications addressing power and resource constraints (while no data is transmitted to the cloud).

**Figure 2:**
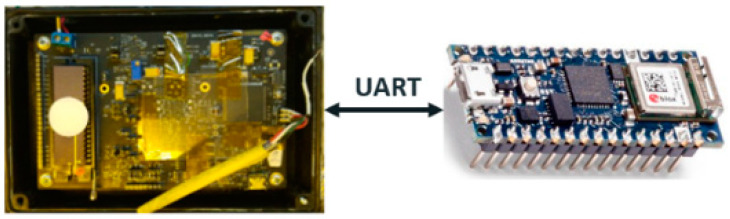
A schematic depiction. An Arduino controller requesting samples from GMOS over UART.

To improve GMOS ability of gas analysis (in real-time), which includes both gas types, as well as gas concentrations, we added a pre-trained neural network, which is written to an Arduino controller [19]. The system is shown in Figure 2.

The neural network was trained using GMOS measurements, mainly the voltage measurements, of different gas types (acetone and ethanol). UART (universal asynchronous receiver transmitter) is used to communicate between the Arduino controller and GMOS. The process includes the controller requesting data from GMOS, data pre-processing, and feature extraction (which later are transmitted as inputs to the neural network). Having a preprocessing data block is crucial, since measurements are always relative to a reference voltage (prior to gas insertion). As a result, we prefer to make sure that all raw data has an equal initial voltage, thus improving the performance of our system.

The preliminary neural network includes three classes (possible gas classifications):

- Ethanol—100 PPM
- Acetone—100 PPM
- None of above (no gas was inserted)

**Figure 3:**
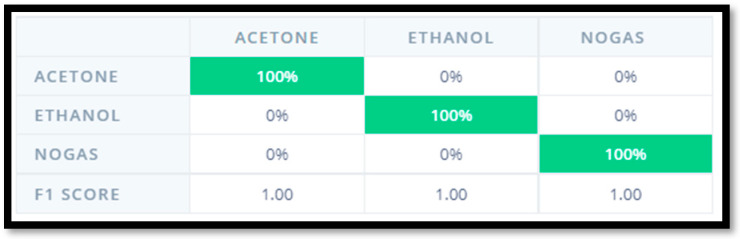
The confusion matrix. Using tinyML, we were able to correctly classify the test set.

For this task, we achieved 100% success on our test set, as our samples were perfectly classified that may be seen in Figure 3. For our system, the current added latency is 1 ms.

We are currently expanding the system, adding more gas types (also as mixtures, as was shown before), as well as different concentrations. 

## 6. Conclusions

This paper presents GMOS’s ability to detect selectively different gases while being updated by more algorithms to enable better real-time analysis of gases. This ability, selectivity, differs GMOS from other sensors in the market. The GMOS technology, CMOS-SOI-MEMS, enables low-power and low-cost operation while maintaining high accuracy. The ability to detect very low gas concentrations (sub-ppm) is also achieved by the implementation of the suspended MEMS-TMOS transistor for detection. As GMOS is under extensive development, more algorithms are being added to improve the sensor’s performance. Both the Kalman filter and the tinyML, which were presented in the article, enable better real-time gas analysis performance. The combination of the CMOS-SOI-MEMS technology with advanced algorithms and AI/ML techniques will improve the performance of the GMOS sensors.

## Data Availability

Not applicable.

## Appendix A

**Table A1:** Performance comparison of different kinds of sensors for ethylene and acetone detection.

Parameter	GMOS	SPG40	ZMOD4410	MiniPID 2
Size [mm^3^] (without evaluation board)	2.2 × 1.6 × 0.3	2.44 × 2.44 × 0.85	3.0 × 3.0 × 0.7	~20 × 20 × 16
Technology	CMOS-SOI-MEMS	MOX (metal oxide)	MOX (metal oxide)	PID (photoionization detector)
Minimum detection limit	20 [ppb]	0.3 [ppm]	160 [ppb]	0.5 [ppb]
Range	~5000 [ppm]	1000 [ppm]	1000 [ppm]	~4000 [ppm]
Selectivity	Selectively ^3^	TVOC ^2^	TVOC	TVOC
Supply voltage [V]	5	3.3	1.7–3.6	3–3.6
Power consumption	20 [mW]	~8.5 [mW]	Down to 160 [μw]	100 [mW] ^1^
Response time [s]	<10	10–30	10	<3
Price	Under development	~10$	~4.3$	1150$
Typical applications	Air quality, fruit refreshment diagnostics, breath control	Indoor air quality	Indoor air monitoring, detection of hazardous materials	Air quality, monitoring

^1^ At 3.3 [V]; ^2^ Total Volatile Organic Compounds (non-selective), ^3^ Selectively detects various gases, such as: Ethanol, Acetone, Ethylene, CO, NH_3_ (also as mixtures).

## References

[1] Nemirovsky Y., Stolyarova S., Blank. T., Bar-Lev S., Svelitza A., Zviaginstev A., Brouk I. (2018). A New Pellistor-Like Gas Sensor Based on Micromachined CMOS Transistor. IEEE Trans. Electron. Devices.

[2] Shlenkevitch D., Avraham M., Stolyarova S., Blank T., Nemirovsky Y. Catalytic Gas Sensor Based on Micro Machined CMOS Transistor. Proceedings of the International Conference on Microwaves, Antennas, Communications and Electronic Systems IEEE COMCAS 2019.

[3] Shlenkevitch D., Avraham M., Stolyarova S., Blank T., Nemirovsky Y. The GMOS—Towards a Gas Sensor for Smart Phones. Proceedings of the 2nd Annual Conference Chemical Sensors for Wearable Devices.

[4] Shlenkevitch D., Stolyarova S., Blank T., Brouk I., Nemirovsky Y. (2020). A Novel Miniature and Selective Combustion Type CMOS Gas Sensor for Gas Mixture Analysis—Part 1: Emphasis on Chemical Aspects. Micromachines.

[5] Avraham M., Stolyarova S., Blank T., Bar-Lev S., Golan G., Nemirovsky Y. (2020). A Novel Miniature and Selective CMOS Gas Sensor for Gas Mixture Analysis—Part 2: Emphasis on Physical Aspects. Micromachines.

[6] Shlenkevitch D., Stolyarova S., Nemirovsky Y. Reducing Food Waste with a Tiny CMOS-MEMS Gas Sensor, Dubbed GMOS. Proceedings of the 7th International Electronic Conference on Sensors and Applications.

[7] SGP40 Datasheet. https://sensirion.com/products/catalog/SGP40/.

[8] ZMOD4410 Datasheet. https://www.renesas.com/us/en/products/sensor-products/environmental-sensors/digital-gas-sensors.

[9] MiniPID 2 Datasheet. https://ionscience.com/en/products/minipid-2-ppm-gas-sensor/.

[10] Tharsika T., Thanihaichelvan M., Haseeb A.S.M.A., Akbar S.A. (2019). Highly Sensitive and Selective Ethanol Sensor Based on ZnO Nanorod on SnO_2_ Thin Film Fabricated by Spray Pyrolysis. Front. Mater..

[11] Shoorangiz M., Shariatifard L., Roshan H., Mirzaei A. (2021). Selective ethanol sensor based on α-Fe_2_O_3_ nanoparticles. Inorg. Chem. Commun..

[12] Grewal M., Andrews A. (2015). Kalman Filtering—Theory and Practice Using MATLAB.

[13] McGrath M.J., Scanaill C.N. (2013). Sensor Technologies—Healthcare, Wellness and Environmental Applications.

[14] Haykin S. (2001). Kalman Filtering and Neural Networks.

[15] Cooper W.S. (1986). Use of Optimal Estimation Theory, in Particular the Kalman Filter, in Data Analysis and Signal Processing.

[16] Luiz G. (2018). Kalman Filters Theory for Advanced Applications.

[17] KalmanFilter. https://www.kalmanfilter.net/default.aspx.

[18] Tiny M.L. https://www.tinyml.org/.

[19] Krayden A., Schohet M., Shmueli O., Shlenkevitch D., Blank T., Stolyarova S., Nemirovsky Y. CMOS-MEMS Gas Sensor Dubbed GMOS for SelectiveAnalysis of Gases with Tiny Edge Machine Learning. Proceedings of the 9th International Electronic Conference on Sensors and Applications.

[20] Yizhe L. Advantages of CMOS Technology in Very Large Scale Integrated Circuits. Proceedings of the 2021 2nd International Conference on Artificial Intelligence in Electronics Engineering (AIEE 2021).

[21] EETimes MEMS Market to Top $22 Billion by 2018. https://www.eetimes.com/mems-market-to-top-22-billion-by-2018/.

[22] Low-Power, High-Sensitivity Infrared Sensor for Presence and Motion Detection. https://www.st.com/content/st_com/en/products/mems-and-sensors/infrared-ir-sensors/sths34pf80.html#overview.

[23] Blank T., Brouk I., Bar-Lev S.H., Amar G., Meimoun E., Meltsin M., Bouscher S.H., Vaiana M., Maierna A., Castagna M.E. (2020). Non-Imaging Digital CMOS-SOI-MEMS Uncooled Passive Infra-Red Sensing Systems. IEEE Sens. J..

[24] Vasilescu G. (2005). Electronic Noise and Interfering Signals.

